# Seismic Behavior and Force-Displacement Characterization of Neotype Column-Slab High Piers

**DOI:** 10.1155/2014/653247

**Published:** 2014-04-22

**Authors:** YanQun Zhou, YeZhi Zhang, MeiXin Ye, MengSi Zhan

**Affiliations:** School of Civil Engineering, Central South University, Changsha 410075, China

## Abstract

The seismic behavior and plasticity spreading of a neotype column-slab high pier are researched in this paper. Four scale model tests of a web slab with two boundary columns are carried out under cyclic inelastic lateral displacements simulating seismic response. The test results show that the neotype column-slab high pier has strong and stable bearing capacity, good ductility, and energy dissipation capacity. The experimental values pertaining to the spread of plasticity are derived. An approach for deriving the spread of plasticity analytically is deduced and applied to the four tests. This method accurately assesses a pier's spread of plasticity for most ductility levels. At nearly all ductility levels, the mean difference between analytical assessments of the spread of plasticity and results from 4 large-scale tests is 12% with a 9% coefficient of variation.

## 1. Introduction

The highway and railway in the western region of China often pass through the gorge region with extremely complex geomorphology and high earthquake intensity [1], so long span bridges with high piers are unavoidable. In the seismic design of piers of bridge substructures, the potential plastic hinge regions need to be carefully detailed for ductility in order to ensure that the shaking from large earthquakes will not cause collapse. In seismic regions, bridges are typically detailed according to capacity design principles, and the piers which support such bridges are designed as ductile members to deform inelastically under a large earthquake without losing strength [2, 3]. Furthermore, the displacement capacity of a bridge as a whole is often assumed to depend heavily on the displacement capacities of individual piers supporting the bridge. According to the recent research, designers have put forward a neotype column-slab high pier [4]. The pier consists of four highly confined corner columns which are connected by slabs and a small amount of beams. The columns provide the pier with inelastic deformation capacity, while slabs and beams provide the pier with stiffness and strength.

Based on a long-span continuous rigid frame bridge with neotype column-slab high piers in the western region of China [4], four scale model tests of a web slab with two boundary columns were designed to investigate the deformation capacity and web crushing failure model of neotype column-slab high piers under cyclic inelastic lateral displacements [5, 6].

Commonly, moment-curvature analyses are the basis of assessing the nonlinear force-displacement response of such a particular reinforced concrete bridge. These analyses need assumptions about the spread of plasticity to calculate plastic rotations and displacements. The concept of the equivalent plastic hinge length, *l*_*p*_, is the base of assessing plastic rotation. The method has a great effect on ductile seismic design. A given plastic curvature is assumed to be lumped in the center of the plastic hinge in this method. If the plastic curvature as a constant is assumed, it is integrated over the plastic hinge length to solve the total plastic rotation.

In addition to the plastic hinge length *l*_*p*_, *l*_*pr*_ is also referred to the length of plastic hinge region. The length of plastic hinge region, *l*_*pr*_, is the physical length over which plasticity actually spreads, which only refers to the length along the pier and does not take into account any penetration of inelastic strain into the footing. It is reasonable to consider that *l*_*pr*_ is not equal to *l*_*p*_. *l*_*p*_ should be proportional to *l*_*pr*_. Researchers have found the effects of three distinct phenomena on the spread of plasticity in reinforced concrete structural components: tension shift, moment gradient, and strain penetration [5]. Although each of those individual effects is well-established, there has been little resolution on the appropriate combination of these effects for assessing the spread of plasticity.

A few equations that include the combined effects of moment gradient and tension shift on the spread of plasticity in reinforced concrete structural components have been proposed [7]. But they have received little attention in the area of bridge design. Until recently, researchers have tried to take into account tension shift and the influence of transverse reinforcement to assess the plastic hinge length of bridge piers. This paper does the same to the neotype column-slab high pier.

Tests are carried out to research the seismic behavior of neotype column-slab high pier in this paper. The experimental values pertaining to the spread of plasticity are derived. Then a method for deriving the spread of plasticity analytically is introduced and applied to four various large-scale neotype column-slab pier tests.

## 2. Experimental Study

Based on a bridge with the height of 112 m of the main pier, four scale model tests of a web slab with two boundary columns were designed. Parameters of the specimens include axial force ratio, web thickness, and cyclic loading regime.

### 2.1. Test Setup

**Figure 1:**
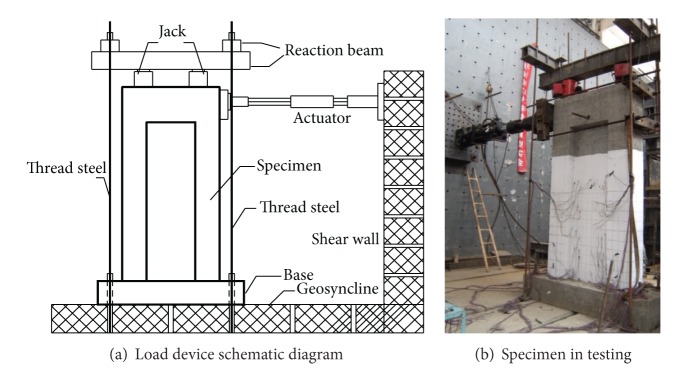
Test setup.

The test setup, designed to load the specimen in-plane as a cantilever, was standard for all of the tests discussed in this paper. The specimens were loaded quasi-statically according to an incrementally increasing, fully-reversed cyclic loading pattern, with constant axial load. The test setup is shown in Figure 1. This particular loading history, in its simplicity, allows for comparison of damage and performance at specified displacement ductility levels.

Referring to* Specification of testing methods for earthquake resistant building*, two cyclic loading regimes were designed for the four specimens (Figure 3) [8]. A few initial cycles were run in load control up to theoretical first yield of the extreme longitudinal reinforcement. The remainder of the tests was conducted in displacement control until failure of the specimens. Only one cycle was applied at each displacement level in Regime A, while each displacement level was cycled two or three times in Regime B. The four specimens were subjected to such constant axial forces and cyclic lateral loading as shown in Table 2.

**Figure 2:**
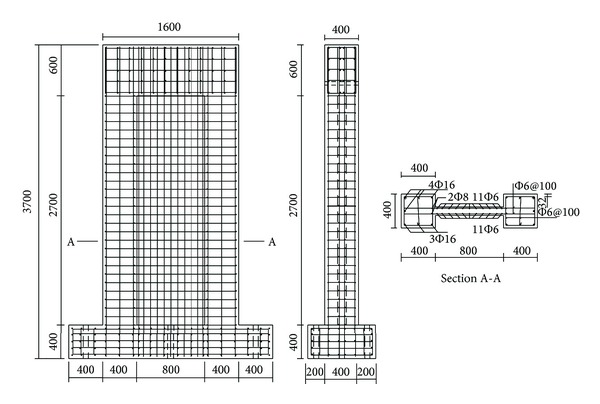
Reinforcement of specimens (dimensions in mm).

**Figure 3:**
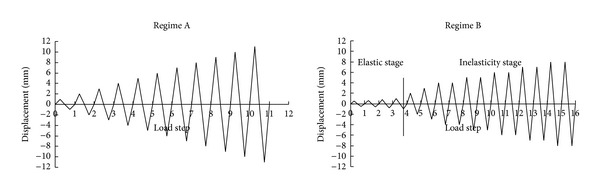
Loading history of specimens.

The reinforcement of the specimens is illustrated in Figure 2. The instrumentation of the specimens included displacement transducers, force transducers and electrical resistance strain gages, allowing us to monitor in-plane displacements, loads, and strains on the longitudinal reinforcement and the concrete surface, respectively.

## 3. Test Results

Until failure, the four specimens exhibited stable hysteretic behavior with minimal pinching. Their flexibility in shear contributed significantly to their overall initial flexibility. After reaching their ideal yield displacement *μ*_Δ_ = 1, furthermore, the specimens continued to gain strength up through *μ*_Δ_ = 2~3 maintaining enough shear stiffness to develop some strain hardening in the longitudinal reinforcement.

**Figure 4:**
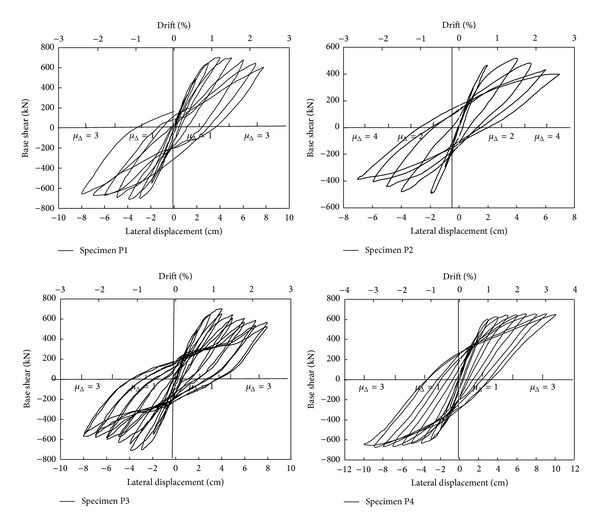
Force-displacement relationships observed in static cyclic tests.

Figure 4 presents hysteretic force-displacement results of the four tests [9, 10]. Besides the observed responses, the plots also include the corresponding values of drift and ductility.

The specimens failed due to the increase of applied displacements. The ultimate displacement was restricted by shear web crushing failure (SF) or flexural-shear web crushing failure (FSF) [11, 12]. The occurrence of SF or FSF depends on the axial force ratio and the web thickness. SF occurred in the elastic range under very high shear stresses. FSF, which occurred under inelastic displacement demands, was concentrated inside the plastic hinge region at the interface of the slab with the boundary column.

The strength, top lateral displacements, achieved ductility and, failure modes are shown in Table 3. Failures of the specimens are assumed to occur either when the shear capacity falls below 85% of the maximum shear capacity, or when the concrete strain in compression reached the ultimate value (*ε*_*u*_ = 0.0038). The ductility is then computed by the ultimate displacement and the extrapolated displacement at first yield (*μ*_Δ_ = Δ_*u*_/Δ_*y*_) (Table 1) [13].

The drift capacity of all specimens is greater than 2%. The test results for specimens P1 and P2 indicate that the higher the axial force ratio, the lesser the drift capacity. Because of the concentration of deformations in the middle of slab crack, the drift capacity of the specimen P1 is the smallest. This concentration depends on the axial force ratio.

## 4. Plastic Hinge Length 

Widely adopting of the plastic hinge length has centered primarily on (1) developed to model the behavior of simple circular and rectangular reinforced bridge piers

(1) $$\begin{array}{c} l_{p}=0.08L+0.022d_{b}f_{y}⩽0.044d_{b}f_{y}\;\;\text{MPa}, \end{array}$$

where *L* = *M*/*V* = member shear span; *d*_*b*_ = longitudinal bar diameter; and *f*_*y*_ = longitudinal bar yield stress. The above equation explicitly takes strain penetration and moment gradient effects into account and is typically applied in conjunction with ultimate curvatures that are limited according to the lower value from

(2) $$\begin{array}{c} \phi_{u}\leq \frac{\left|\varepsilon_{c}\right|+\left|\varepsilon_{s}\right|}{D^{\prime }},\\ \phi_{u}\leq \frac{\varepsilon_{cu}}{c}, \end{array}$$

where *ε*_*s*_ = extreme steel fiber strain; *ε*_*c*_ = extreme confined concrete fiber strain from moment-curvature analysis; *D*′ = distance between *ε*_*c*_ and *ε*_*s*_; *ε*_*su*_ = strain of the longitudinal reinforcement at ultimate stress; *ε*_*cu*_ = ultimate concrete strain; and *c* = distance from the neutral axis to the extreme confined concrete compression fiber. Because the date, on which (1) was based, implied that the effects of tension shift were statistically insignificant, the equation has no tension shift component.

Experimental results from the four large-scale tests based on the neotype column-slab high pier have distinguished the three components of the plastic hinge length: tension shift, moment gradient, and strain penetration. However, during the course of calculation of the experimental plastic hinge length for each test, the inherent difficulties have been exposed in calculating experimental values such as the base curvature and the plastic hinge length.

The experimental values pertaining to the spread of plasticity are derived. At the same time, this paper presents an approach for deriving the spread of plasticity analytically for all levels of displacement ductility in the four members. This method has been proven effective for such highly complicated bridge piers.

### 4.1. Experimental Plastic Hinge Length

**Figure 5:**
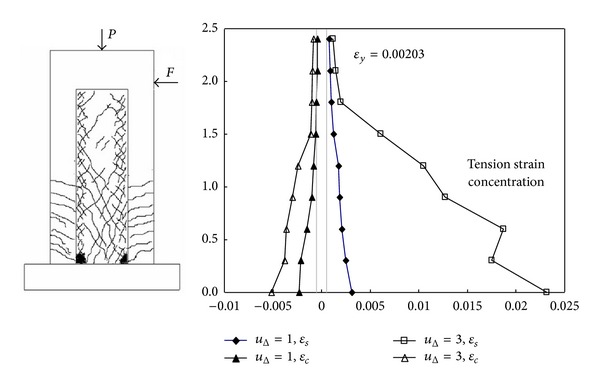
Comparison of crack pattern with experimental strain distributions of Specimen P4.

A concentration of tension strains engenders at the section of maximum moment. This section is assumed to be at the base of a column. This concentration complicates the notion of base curvature and increases the difficulty in calculating flexural deformations according to actual strain levels at the column base.  Figure 5 compares the experimental strain distributions of Specimen P4 along the column height, at a displacement ductility (*u*_Δ_ = Δ/Δ_*y*_) of 3 [14], with the crack pattern of Specimen P4 in the plastic hinge region, which shows the linear potentiometers required by (3).

**Figure 6:**
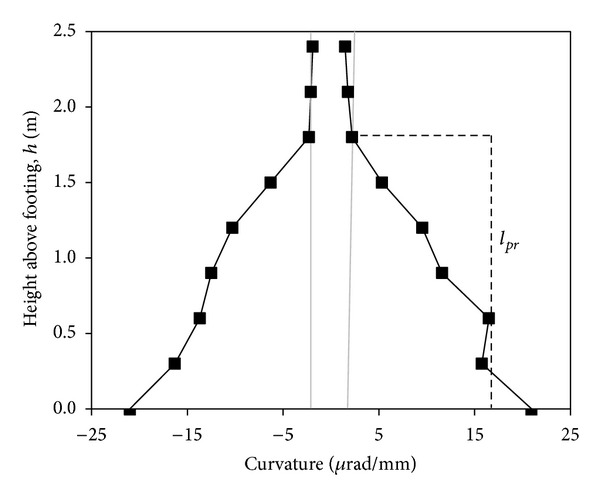
Specimen P4, curvature *μ*_Δ_ = 3.

The experimental curvatures reported in this paper are calculated as

(3) $$\begin{array}{c} \phi =\frac{\Delta_{t}-\Delta_{c}}{D_{\phi }L_{g}}=\frac{\varepsilon_{s}^{\prime }-\varepsilon_{c}^{\prime }}{D^{\prime }}, \end{array}$$

here Δ_*t*_ = elongation of a linear potentiometer on the tension size of the specimen (a positive number), and Δ_*c*_ = shortening of a linear potentiometer on the compression side of the specimen at the same horizontal level (a negative number). Both potentiometers have the same gage length *l*_*g*_. *D*_*ϕ*_ = distance between the two linear potentiometer. *ε*_*s*_′ and *ε*_*c*_′ are steel and concrete strains calculated from potentiometer readings. *D*′ = distance between extreme fiber steel and confined concrete strains.  Figure 6 shows the curvature distributions derived according to (3) for both the pull and push directions.

Zatar and Mutsuyoshi [15] proposed that, if the plastic curvatures were assumed to be linearly distributed along a certain portion of the column height, the experimental plastic hinge length could be assessed. Further observation of experimental data has shown consistently that plastic curvatures follow an approximately linear distribution inside the plastic hinge region [16]. Assuming that plastic curvature is linearly distributed from the column base up to a height of *l*_*pr*_, and that plastic rotation occurs primarily about the column base, *l*_*p*_ can be evaluated as

(4) $$\begin{array}{c} l_{p}=\frac{\Delta_{pf}}{\phi_{p}H}=\frac{l_{pr}}{2}+l_{sp}, \end{array}$$

where Δ_*pf*_ = plastic flexural displacement and *ϕ*_*p*_ = plastic curvature. The plastic flexural displacement is expressed to calculate experimental plastic curvature as follows:

(5) $$\begin{array}{c} \Delta_{pf}=\Delta -\Delta_{s}-\Delta_{yf}^{\prime }\frac{M}{M_{y}^{\prime }}, \end{array}$$

where Δ = total experimental displacement; Δ_*s*_ = experimental shear displacement; Δ_*yf*_′ = experimental flexural displacement at first yield; and *M*_*y*_′ = theoretical first yield moment, and *M* = moment at the column base calculated as *M* = *FH*, where *F* = measured actuator lateral force. The plastic curvature is expressed as

(6) $$\begin{array}{c} \phi_{p}=\phi_{b}-\phi_{y}^{\prime }\frac{M}{M_{y}^{\prime }}, \end{array}$$

where *ϕ*_*b*_ = base curvature and *ϕ*_*y*_′ = theoretical first yield curvature.

### 4.2. Assessing Spread of Plasticity Analytically

**Figure 7:**
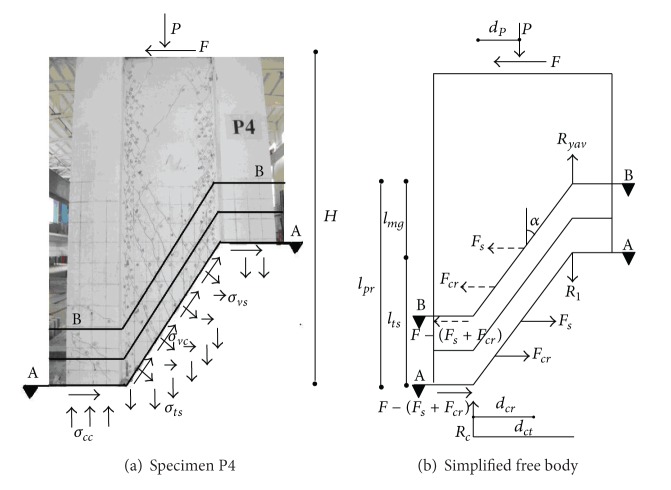
Free body diagrams for evaluating spread of plasticity in neotype column-slab pier.

Equations that depict the spread of plasticity in bridge piers are derived from Section A-A showed in Figure 7. The equations are based on parameters that can be depicted from moment-curvature analyses. In this research, the moment-curvature analyses explain nonlinear material behavior of longitudinal reinforcement, confined and unconfined concrete in compression according to Mander et al. [17], and tension stiffening in the confined and unconfined concrete according to Hines et al. [18].

Figure 7 characterizes two free-body diagrams and the most important parameters influencing the spread of plasticity. In Figure 7(a), Specimen P4 is shown with the portion below Section A-A cutaway. The specimen is kept in equilibrium with *σ*_*vc*_ = principal tensile stresses and aggregate interlock stresses along the surface of the concrete; *σ*_*cc*_ = flexural compressive stresses in the compression steel and concrete; *σ*_*vs*_ = horizontal tensile stresses in the transverse reinforcement; and *σ*_*ts*_ = flexural tensile stresses in the longitudinal reinforcing steel.  Figure 7(b) shows a simplified free-body diagram cut on Section A-A. In this diagram, the flexural compressive force resultant equals *R*_*c*_, and the vertical flexural tensile stresses in the concrete and in the longitudinal reinforcing steel are concentrated into the tensile force resultant *R*_1_. The distance between the flexural tensile concrete force resultant *R*_*cr*_ (included in the parameter *R*_1_) and the compressive force resultant *R*_*c*_ equals *d*_*cr*_. The horizontal length *d*_*p*_ measures the distance between the compressive force resultant *R*_*c*_ and the axial load *P*. The distance between the compression and tension resultants equals *d*_*ct*_. At the column base, the flexural tensile force resultant *R* and the compressive force resultant *R*_*c*_ are the only internal forces.

Assuming that the parameters above are acquirable as output from a moment-curvature analysis for any level of curvature, the moment of resistance at the base of the column can be calculated exactly as

(7) $$\begin{array}{c} M=FH={Rd}_{ct}+{Pd}_{p}. \end{array}$$

Further assumptions made for the following derivation are shown in Figure 7(b). The crack angle at Section A-A is inclined at an angle *α* from vertical and extends straight from the compression resultant *R*_*c*_ to the flexural tension resultant *R*_1_, reaching a height of *l*_*ts*_ = *d*_*ct*_ cot *α*. The tensile stress carried by the transverse reinforcement *σ*_*vs*_ is assumed to be concentrated at a height of *d*_*ct*_ cot *α*/2(*l*_*ts*_/2) as the resultant horizontal force *F*_*s*_. Moreover, the horizontal tensile stresses in the concrete are assumed to be concentrated into the resultant horizontal force *F*_*cr*_ at a distance *d*_*cr*_ cot *α* above the base of the specimen.

The value *R*_*yav*_ is applied as the effective flexural tensile yield force resultant

(8) $$\begin{array}{c} R_{yav}=\frac{R_{y}^{\prime }+R_{y}}{2}, \end{array}$$

where *R*_*y*_′ = flexural tensile force resultant at first yield of the extreme steel and *R*_*y*_ = flexural tensile force resultant when either *ε*_*s*_ = 0.015 or *ε*_*c*_ = 0.0038 is first reached. Assuming that *R*_1_ = *R*_*yav*_, and that the stress on Section A-A in Figure 7(a) can be approximated by the resultant forces in Figure 7(b), the moment equilibrium gives

(9) $$\begin{array}{c} \left(R-R_{yav}\right)d_{ct}-\left(F_{s}+F_{cr}\frac{2d_{cr}}{d_{ct}}\right)\frac{d_{ct}\;\text{cot}\;\alpha }{2}=0. \end{array}$$

Assuming that all transverse steel in the plastic hinge region has yielded, *σ*_*vs*_ can be expressed as

(10) $$\begin{array}{c} \sigma_{vs}=\frac{A_{v}\sigma_{yv}}{s}d_{ct}\;\text{cot}\;\alpha , \end{array}$$

where *A*_*v*_ = area of transverse reinforcement at a given level, *σ*_*yv*_ = yield stress of the transverse reinforcement; *s* = space between the transverse bars. Table 2 gives these values for the four specimens.

The horizontal concrete tensile force resultant *R*_*cr*_ is calculated based on values originated from moment-curvature analysis explained as follows. The average flexural tensile concrete stress *σ*_*tr*_ is estimated as

(11) $$\begin{array}{c} \sigma_{tr}=\frac{R_{cr}}{\left(D-d\right)t_{w}}, \end{array}$$

where *t*_*w*_ = effective width of the section, *D* = member total section depth, *d* = neutral axis depth. Principal tensile stress in the concrete *σ*_1_ is related to horizontal and vertical tensile concrete stresses according to a 45° shear crack angle. Therefore,

(12) $$\begin{array}{c} \sigma_{1}={1.4\sigma }_{tr}={1.4\sigma }_{cr}, \end{array}$$

where *σ*_*cr*_ = horizontal tensile concrete stress. Based on (11) and (12), *F*_*cr*_ is proximate in a form similar to (10)

(13) $$\begin{array}{c} F_{cr}=\sigma_{cr}t_{w}d_{ct}\;\text{cot}\;\alpha , \end{array}$$

where *σ*_*cr*_ *t*_*w*_ = average distributed horizontal tensile force in the concrete.

Combing (9), (10), and (13) results in

(14) $$\begin{array}{c} l_{pr}=d_{ct}\;\text{cot}\;\alpha =\sqrt{\frac{2\left(R-R_{yav}\right)d_{ct}}{\left(A_{v}\sigma_{yv}/s+\sigma_{cr}t_{w}\left(2d_{cr}/d_{ct}\right)\right)}}. \end{array}$$

Equation (14) describes the effect of tension shift on the spread of plasticity, but it only partly describes the effect of moment gradient. The expression (*R* − *R*_*yav*_) captures the difference between the yield moment and values above the yield moment, but it does not capture the possible spread of plasticity into a shear span's uniform diagonal stress field. This spread depends on the length of the shear span *H*. To predict such spread of plasticity by (4), the crack angle marking the end of the stress field in the plastic hinge region would have to reach *α*< 15°. In response to such case, the crack angle should be limited by the estimated shear crack behavior of the column. According to (14), plasticity is assumed to spread only until the slop of compression struts corresponds to cot *α*_1_, where *α*_1_ = crack angle limited not according to spreading plasticity, but according to shear. Just as shown in Figure 7, the flexural tensile force resultant *R*_1_ at this level is therefore no longer equivalent to the effective tensile yield force *R*_*yav*_. Based on (9), (10), and (13), *R*_1_ can be estimated as

(15) $$\begin{array}{c} R_{1}=R-\frac{d_{ct}\;{\text{cot}}^{2}\alpha_{1}}{2}\left(\frac{A_{v}\sigma_{yv}}{s}+\sigma_{cr}t_{w}\frac{2d_{cr}}{d_{ct}}\right)\geq R_{yav}. \end{array}$$

The diagonal crack angle *α*_1_ can be estimated as in the following equation according to Mander et al. [17]:

(16) $$\begin{array}{c} \alpha_{1}={\text{cot}}^{-1}\left(\frac{F}{\left(A_{v}\sigma_{yv}/s+\sigma_{1}t_{w}\right)d_{ct}}\right)<90^{{}^{\circ}}, \end{array}$$

where *σ*_1_ = 1.4*σ*_*tr*_. Combining the assumption with the observation that (2*d*_*cr*_/*d*_*ct*_) ≈ 1.4 for all four specimens allows (15) and (16) to be combined very simply as

(17) $$\begin{array}{c} R_{1}=R-F\frac{\text{cot}\;\alpha_{1}}{2}\geq R_{yav}. \end{array}$$

Considering the free-body diagram between Section A-A and B-B in Figure 7, where relevant equilibrating vertical and horizontal forces on the top portion are shown as dashed arrows, *l*_*mg*_ can be answered as (18) based on moment equilibrium. Consider

(18) $$\begin{array}{c} l_{mg}=\left(R_{1}-R_{yav}\right)\frac{d_{ct}}{F}\geq 0. \end{array}$$

Further combining (16) to (18) allows for *l*_*pr*_ to be calculated as

(19) $$\begin{array}{c} l_{pr}=\left(R-R_{yav}\right)\frac{d_{ct}}{F}+\frac{F}{2\left(A_{v}\sigma_{yv}/s+\sigma_{1}t_{w}\right)}\geq 0. \end{array}$$

### 4.3. Comparison of Experimental and Analytical Results

**Figure 8:**
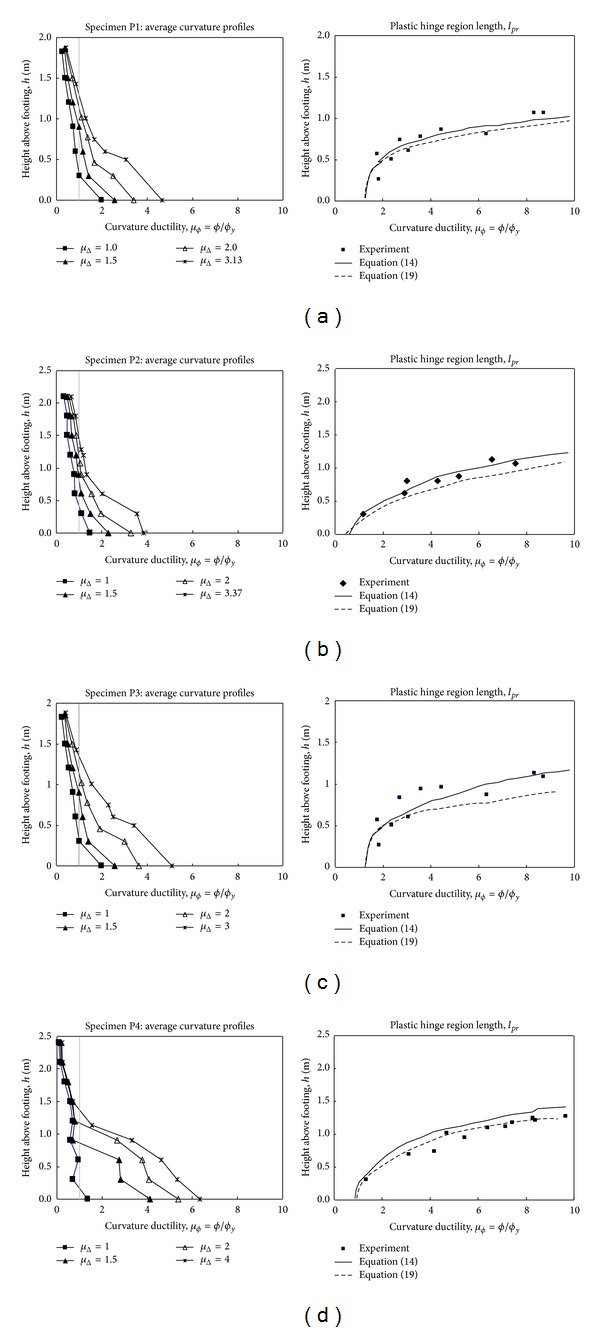
Experimental and theoretical spread of plasticity, *l*_*pr*_.

Figure 8 compares (14) (bond stress model) and (19) (shear crack model) with the experimental results, which shows that both equations can adequately predict the spread of plasticity at most levels of *u*_*ϕ*_. Both the theories and the experiment show plasticity spreading further with increasing curvature ductility. They also show plasticity spreading rapidly between *μ*_Δ_ = 1 and *μ*_Δ_ = 2 and then slowing down for higher levels of displacement ductility.

Equation (14) provides more accurate results than (19) (refer to Figures 8(a) to 8(c)). For thinner web slab with boundary elements such as Specimen P4, (19) provides more accurate results than (14) (refer to Figure 8(d)).

Table 4 compares *l*_*pr*_ and *l*_*p*_ of the two models with experimental results. Column (1) identifies the specimens. Columns (2) to (9) report the mean differences between experiment and analysis and their coefficients of variation (COV) for the values *l*_*pr*_ ((14) and (19)), *l*_*p*_ (4). Mean differences between analysis and experiment are reported for all ductility levels ranging from *μ*_Δ_ = 1 to failure of the test specimens. The mean difference for all of the specimens combined is 12% with a 9% COV (Table 4, (19)).

## 5. Conclusions

1. The displacement ductility factors of all specimens are between 3.0 and 4.0, which shows that the neotype column-slab high pier has good ductility and seismic performance. The drift capacity of all specimens is greater than 2%, which depends on the axial force ratio and web thickness.
2. The axial force ratio has great effect on the yield load, peak load, and the ultimate displacement. The repeat of the same displacement load accelerates the decline in intensity, reduces the ultimate displacement, and increases the cumulative damage on the plastic region of Specimen P3. The web thickness has a great influence on the seismic behavior of the specimens. Both the yield and ultimate loads of Specimen P4 are smaller than those of Specimen P2. But the bearing capacity of the former reduces rarely and its ductility is relatively good after peak loading.
3. This paper incorporates tension shift and its dependence on transverse reinforcement into the assessment of the plastic hinge length of the neotype column-slab high piers. The approach given by (14) and (19) is adopted to assess the actual physical behavior of bridge piers at all levels of horizontal force and displacement in greater detail and with greater accuracy, which has been proven effective for the neotype column-slab high piers.

## Figures and Tables

**Table 1:** Mechanical properties of steel.

Diameter (mm)	Standard	*f*_*y*_′ N·mm^−2^	*f*_*u*_ N·mm^−2^	*ε*_*y*_ (×10^−6^)	*E*_*s*_ N·mm^−2^
16	HRB335	421.14	538.08	2512	1.924 × 10^5^
10	HPB235	411.2	463.1	2371	1.842 × 10^5^
8	HPB235	351.2	408.0	1913	1.803 × 10^5^
6	HPB235	342.5	396.3	1681	1.791 × 10^5^

**Table 2:** Characteristics of specimens.

Specimen	*d* (mm)	*n *	*N* (kN)	Loading regime
P1	100	0.15	1948	A
P2	100	0.1	1298	A
P3	100	0.15	1948	B
P4	80	0.15	1840	A

**Table 3:** Test results: strength, top lateral displacements, and ductility.

Specimen	Yield		Ultimate			Failure mode
	*P*_*y*_ (kN)	Δ_*y*_ (cm)	*P*_*u*_ (kN)	Δ_*u*_ (cm)	*μ*_Δ_	
P1	604	2.49	604	7.79	3.13	SF
P2	450	1.72	400	6.99	4.06	FSF
P3	604	2.31	598	6.98	3.02	FSF
P4	596	2.98	648	10.05	3.37	FSF

**Table 4:** Mean differences and coefficients of variation for analytical and experimental values of *l*_*pr*_ and *l*_*p*_.

Specimen	Bond stress model (14)				Shear crack model (19)			
	*l*_*pr*_, % difference		*l*_*p*_, % difference		*l*_*pr*_, % difference		*l*_*p*_, % difference	
	Mean	COV	Mean	COV	Mean	COV	Mean	COV
(1)	(2)	(3)	(4)	(5)	(6)	(7)	(8)	(9)
P1	9	14.6	5.5	12	12.8	15	6	15
P2	6	8	8	10	14	9	8	13
P3	11	7	10	12.5	16	8	12	20
P4	12	11	10	14	4	6	6.5	19
All specimens	9.5	10	8	12	12	9	8	17
